# Alteration of faecal microbiota balance related to long-term deep meditation

**DOI:** 10.1136/gpsych-2022-100893

**Published:** 2023-01-03

**Authors:** Ying Sun, Peijun Ju, Ting Xue, Usman Ali, Donghong Cui, Jinghong Chen

**Affiliations:** 1 Shanghai Mental Health Center, Shanghai Jiao Tong University School of Medicine, Shanghai, China; 2 Shanghai Key Laboratory of Psychotic Disorders, Shanghai, China; 3 Shanghai Institute of Traditional Chinese Medicine for Mental Health, Shanghai, China; 4 Department of Pharmacology, Shaheed Zulfiqar Ali Bhutto Medical University, Islamabad, Pakistan

**Keywords:** Psychosomatic Medicine, Mental Health, Healthy Lifestyle

## Abstract

**Background:**

Advancements in research have confirmed that gut microbiota can influence health through the microbiota–gut–brain axis. Meditation, as an inner mental exercise, can positively impact the regulation of an individual’s physical and mental health. However, few studies have comprehensively investigated faecal microbiota following long-term (several years) deep meditation. Therefore, we propose that long-term meditation may regulate gut microbiota homeostasis and, in turn, affect physical and mental health.

**Aims:**

To investigate the effects of long-term deep meditation on the gut microbiome structure.

**Methods:**

To examine the intestinal flora, 16S rRNA gene sequencing was performed on faecal samples of 56 Tibetan Buddhist monks and neighbouring residents. Based on the sequencing data, linear discriminant analysis effect size (LEfSe) was employed to identify differential intestinal microbial communities between the two groups. Phylogenetic Investigation of Communities by Reconstruction of Unobserved States (PICRUSt) analysis was used to predict the function of faecal microbiota. In addition, we evaluated biochemical indices in the plasma.

**Results:**

The α-diversity indices of the meditation and control groups differed significantly. At the genus level, *Prevotella* and *Bacteroides* were significantly enriched in the meditation group. According to the LEfSe analysis, two beneficial bacterial genera (*Megamonas* and *Faecalibacterium*) were significantly enriched in the meditation group. Functional predictive analysis further showed that several pathways—including glycan biosynthesis, metabolism and lipopolysaccharide biosynthesis—were significantly enriched in the meditation group. Moreover, plasma levels of clinical risk factors were significantly decreased in the meditation group, including total cholesterol and apolipoprotein B.

**Conclusions:**

Long-term traditional Tibetan Buddhist meditation may positively impact physical and mental health. We confirmed that the gut microbiota composition differed between the monks and control subjects. The microbiota enriched in monks was associated with a reduced risk of anxiety, depression and cardiovascular disease and could enhance immune function. Overall, these results suggest that meditation plays a positive role in psychosomatic conditions and well-being.

**What is already known on this topic**

- Accumulated evidence suggests that gut microbiota can influence health through the microbiota–gut–brain axis. As an inner mental exercise, meditation can positively impact the regulation of psychosomatic conditions. However, few studies have comprehensively investigated the faecal microbiota following meditation.

**What this study adds**

- Long-term deep meditation may regulate gut microbiota homeostasis and thus affect physical and mental health.

**How this study might affect research, practice or policy**

- We confirmed that long-term deep meditation, represented by Tibetan Buddhism, could positively impact physical and mental health by regulating faecal microbiota. For example, long-term meditation affects human health through the glycan and lipopolysaccharide biosynthesis pathways. Plasma biochemical indices further confirmed that long-term meditation enhanced the body’s immune function and reduced the risk of cardiovascular disease.

## Introduction

Tibetan Buddhist meditation, known to originate from ancient Indian Ayurveda, can be defined as a form of psychological training. This practice is known to exercise the mind and allows self-regulation of the body to cultivate well-being and provide insights into the true nature of all phenomena. Meditation has been used for millennia to achieve wisdom.^1 2^ Meditative exercises regulate attention and emotions, focusing outwards on specific physical and sensory stimuli and turning inwards toward spiritual experiences and the somatic sensation of physical experience.^3^ Meditation is a self-induced process using a specified relaxation technique to gain a state of psychophysical ability that modifies the centre of self-consciousness and self-focus.^4^ Owing to its positive impact on specific areas of psychopathology including depression, anxiety, chronic pain and substance abuse, along with its association with attention disorders, traumatic stress, eating disorders and serious mental illness, meditation is increasingly being incorporated into mental health interventions.^5^ A recent study has shown that meditation affords a protective plasma proteome, can offset obesity and hypertension, and reduce heart rate variability.^6^

The gut microbiota can influence the brain and profoundly impacts mood and behaviour through the microbiota–gut–brain axis.^7 8^ The axis consists of two-way communication between the brain and gut microbiota, and it functions via microbial byproducts, immune and inflammatory pathways, neuroendocrine and enteroendocrine signalling, stress response and the vagus nerve.^9–12^ Given the crucial role of microbiota in human health mediated via the microbiota–gut–brain axis, the mechanism through which long-term deep meditation influences the gut microbiota is of increasing interest. One study has focused on the effect of meditation and a vegan diet on gut microbiota.^13^ However, whether meditation alone affects microbiota composition remains unclear.

In this study, we aimed to investigate the association between traditional long-term Tibetan Buddhist meditation and faecal microbiota and to explore further whether meditation can impact human health by manipulating gut bacteria as a novel target. Samples were collected from monks and neighbouring populations of three temples in Tibet, China, although samples from high-altitude areas were extremely difficult to obtain. To the best of our knowledge, this is the first study examining the faecal microbiota of Tibetan Buddhist monks.

## Methods

### Sample collection

We collected a total of 128 samples. Subsequently, samples whose subjects had taken antibiotics and yoghurt or samples of poor quality were excluded, resulting in 56 eligible samples. Given the extreme sparsity of subjects who qualified as controls in the region, recruitment of adequate numbers was difficult, leading to unequal populations of monks and control subjects. To achieve mind training, Tibetan Buddhist monks performed meditation practices of Samatha and Vipassana for at least 2 hours a day for 3–30 years (mean (SD) 18.94 (7.56) years). Samatha is the Buddhist practice of calm abiding, which steadies and concentrates the mind by resting the individual’s attention on a single object or mantra. Vipassana is an insightful meditation practice that enables one to enquire into the true nature of all phenomena.

Faecal and peripheral venous blood samples were collected, placed in a portable refrigerator at −20 °C, shipped to Shanghai and stored at −80 °C until subsequent processing. The serum was separated by centrifugation at 5 ℃, followed by storage at −20 ℃. Plasma biochemical indices were measured at the Shanghai Mental Health Centre, Shanghai Jiao Tong University School of Medicine. All samples were collected and measured by professionals.

### 16S rRNA gene amplicon sequencing and analysis

Total DNA extraction was performed using the QIAamp Fast DNA Stool Mini Kit (Qiagen, Germany). DNA concentration and purity were determined using a Thermo NanoDrop 2000, and the quality of extracted DNA was validated by performing 1% agarose gel electrophoresis.

The V3–V4 variable region of the bacterial 16S ribosomal RNA (16S rRNA) gene was amplified by polymerase chain reaction (95 °C for 3 min followed by 30 cycles at 98 °C for 20 s, 58 °C for 15 s and 72 °C for 20 s, and a final extension at 72 °C for 5 min) using the relevant primers (341F: 5′-CCTACGGGRSGCAGCAG-3′; 806R: 5′-GGACTACVVGGGTATCTAATC-3′). Amplicons were extracted from 2% agarose gels, purified using the AxyPrep DNAGel Extraction Kit (Axygen Biosciences, California, USA) following the manufacturer’s instructions, and quantified using Qubit2.0 (Invitrogen, California, USA). The normalised equimolar pooled concentrations of each amplicon were sequenced on an Illumina HiSeq PE250 sequencing instrument (Illumina, California, USA). Paired-end reads of 250 bp overlapped on their 3′ ends for concatenation into the original longer tags using PANDAseq (https://github.com/neufeld/pandaseq, version 2.9). The sequencing output was generated as demultiplexed fastq-files for downstream analyses. DNA extraction, library construction and sequencing were performed at the Realbio Genomics Institute (Shanghai, China).

The assembled tags, trimmed barcodes and primers were further checked for their rest lengths and average base quality. The 16S tags were restricted between 220 and 500 bp, such that the average Phred score of bases was ≥20 (Q20) and ≤3 ambiguous N. The tag copy number was enumerated and the redundancy of repeated tags was removed. Only tags with a frequency of ˃1, which is more reliable, were clustered into operational taxonomic units (OTUs), each with a representative tag. The OTUs were clustered with 97% similarity using UPARSE (http://drive5.com/uparse/), and chimeric sequences were identified and removed using Usearch (V 7.0.1090). Each representative tag was assigned to a taxon by the Ribosomal Database Project (RDP) Classifier against the RDP database using a confidence threshold of 0.8. The OTU profiling table and α- and β-diversity analyses were performed using Python scripts of QIIME (version 1.9.1).

### Statistical analysis

An independent sample t-test was used to analyse normally distributed variables. Non-normally distributed variables were analysed using the Mann–Whitney U test. A p value of <0.05 was considered statistically significant. Statistical analysis was performed using GraphPad Prism software (San Diego, California, USA). Analysis of similarities (ANOSIM) was performed using the vegan package in R. Principal coordinates analysis (PCoA) was performed using the ade4 package in R. Linear discriminant analysis (LDA) effect size (LEfSe) was performed using the LEfSe Tools.^14^ Phylogenetic examination of communities was achieved via the reconstruction of unobserved states (PICRUSt) analysis using the PICRUSt software.^15^

## Results

### Participant information and study design

**Figure 1:**
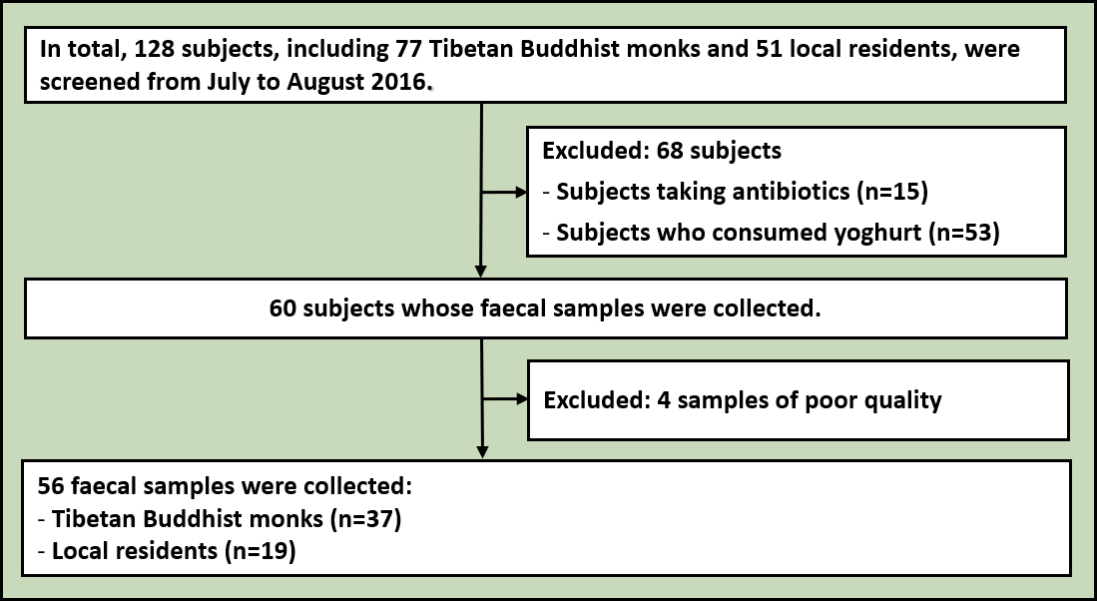
Flowchart of enrolled subjects.

The meditation group consisted of 37 Tibetan Buddhist monks from the Qiongke, Jiaqu and Ezhi Temples, and the control group comprised 19 residents neighbouring the temples (figure 1). No enrolled subject had taken antibiotics, probiotics, prebiotics or antifungal medications for 3 months before faecal sample collection. All subjects were male. No significant differences were observed in age (Mann–Whitney test, p=0.089, U=253.5), systolic blood pressure (Mann–Whitney test, p=0.442, U=306.5), diastolic blood pressure (Mann–Whitney test, p=0.722, U=330.5) or heart rate (Mann–Whitney test, p=0.173, U=249). Moreover, both groups had the same dietary structure. The staple food mainly included highland barley, rice, steamed bread and noodles, and the supplementary food primarily comprised vegetables, meat and butter tea.

### Comparison of structural characteristics of the gut microbiota

**Figure 2:**
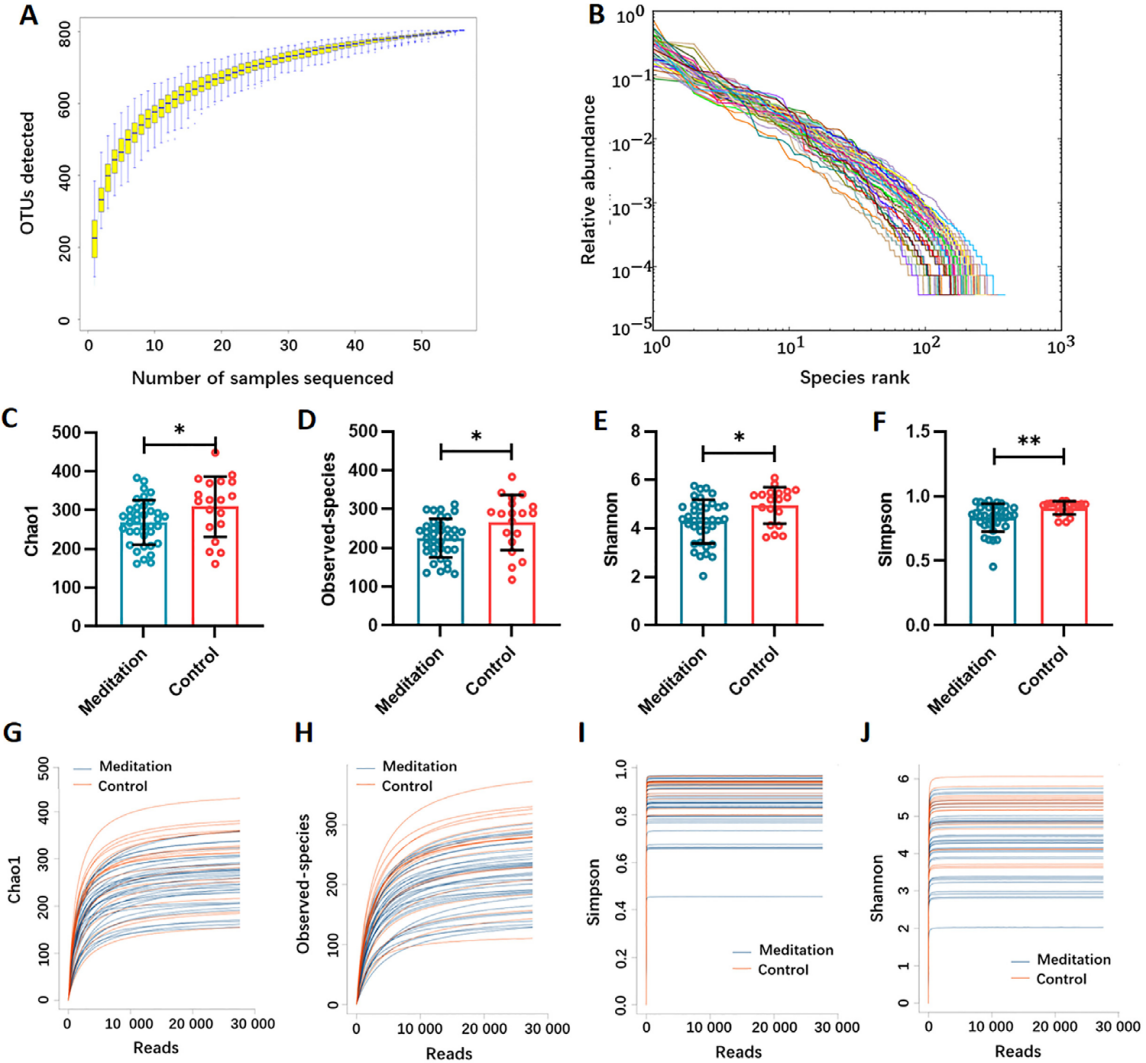
Alpha diversity of the gut microbiota. (A) Species accumulation curve between the number of samples sequenced. (B) The relative bacterial evenness was evaluated by the rank abundance curves. Gut microbial diversity was estimated by the Chao1 index (C), observed-species index (D), Shannon index (E) and Simpson index (F) in the meditation and control groups; Mann–Whitney test, *p<0.05, **p<0.01. Gut microbial dilution curve of Chao1 index (G), observed-species index (H), Shannon index (I) and Simpson index (J) in the meditation and control groups. OTUs, operational taxonomic units.

Gut microbiota was assessed by performing 16S rRNA HiSeq sequencing. The average valid 16S rRNA gene sequence number was 36 210.84 (range 32 872–38 923) for each sample. After the taxonomic assignment, 803 OTUs were identified. The species accumulation curve of all samples reached an asymptote, suggesting the adequacy of our sampling efforts (figure 2A). Additionally, rank abundance curves were employed to evaluate the relative bacterial evenness, exhibiting similar patterns across all samples (figure 2B). Alpha diversity indices (Chao1, observed-species, Shannon and Simpson) were calculated to assess the bacterial diversity per sample using sampling-based OTUs. The results showed that the gut microbial α-diversity was significantly higher in the control group than in the meditation group (Mann–Whitney test, figure 2C–F). The dilution curve analysis based on the α-diversity indices revealed that the sequencing volume covered all micro-organisms in samples and met the data analysis requirements (figure 2G–J).

**Figure 3:**
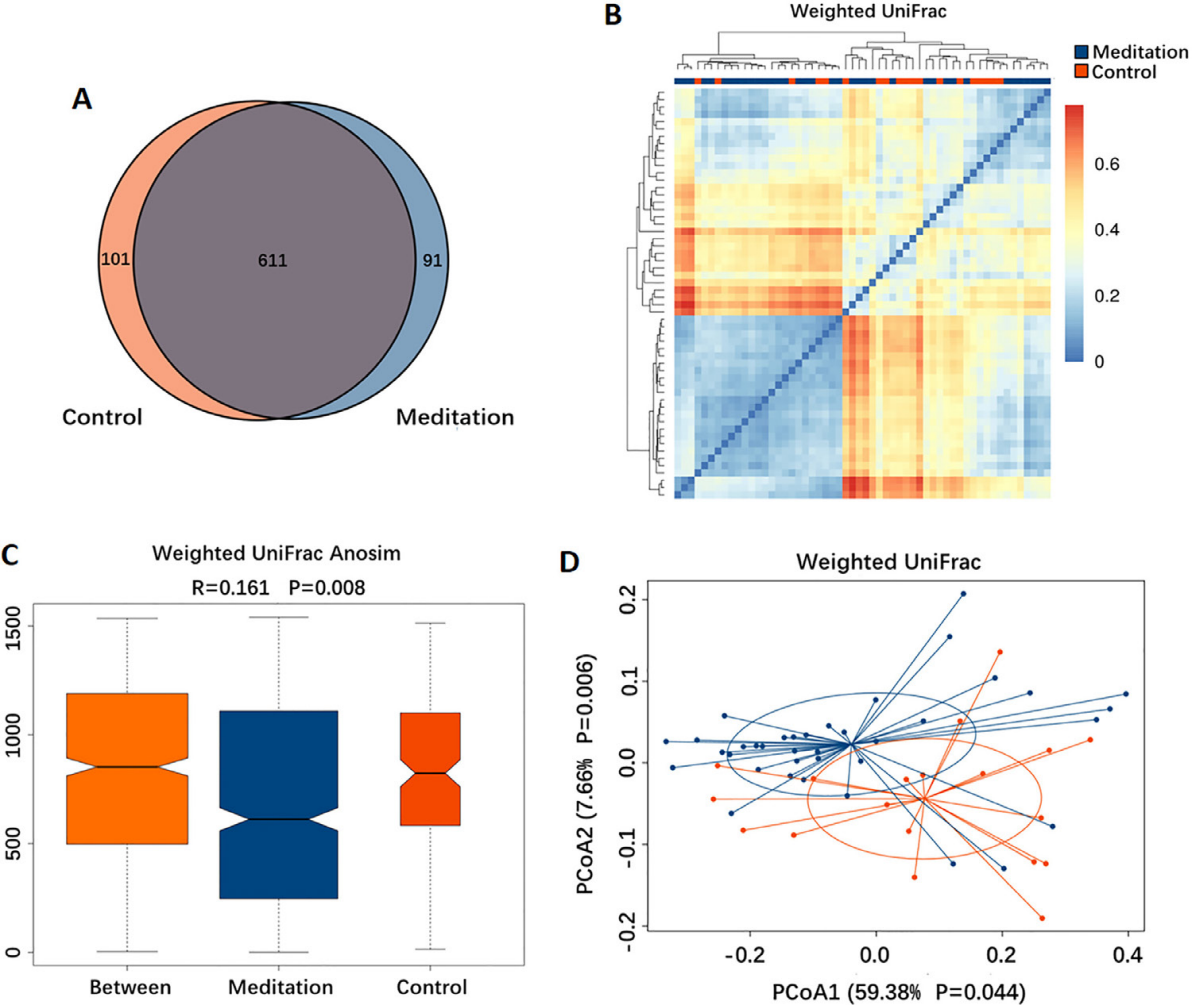
Beta-diversity of the gut microbiota between the meditation and control groups. (A) Venn diagram showing the overlaps between groups. (B) Distance distribution heatmap using weighted UniFrac. (C) Weighted UniFrac analysis of similarities (ANOSIM) analysis. (D) Principal coordinates analysis (PCoA) using weighted UniFrac.

Based on the Venn diagram, 611 out of 803 OTUs were shared between the two groups, whereas 91 and 101 were unique to the meditation and control groups, respectively (figure 3A). To display the microbiome space between samples, we calculated the β-diversity using the weighted UniFrac method, along with a distance distribution heatmap (figure 3B), ANOSIM (figure 3C) and PCoA (figure 3D). Based on the findings of ANOSIM (R=0.161, p=0.008), the difference between groups was significant (figure 3C) and greater than that within groups. The results of the PCoA showed that the gut microbiota in the meditation group clustered separately from that in the control group (figure 3D).

### Screening of different key microorganisms

**Figure 4:**
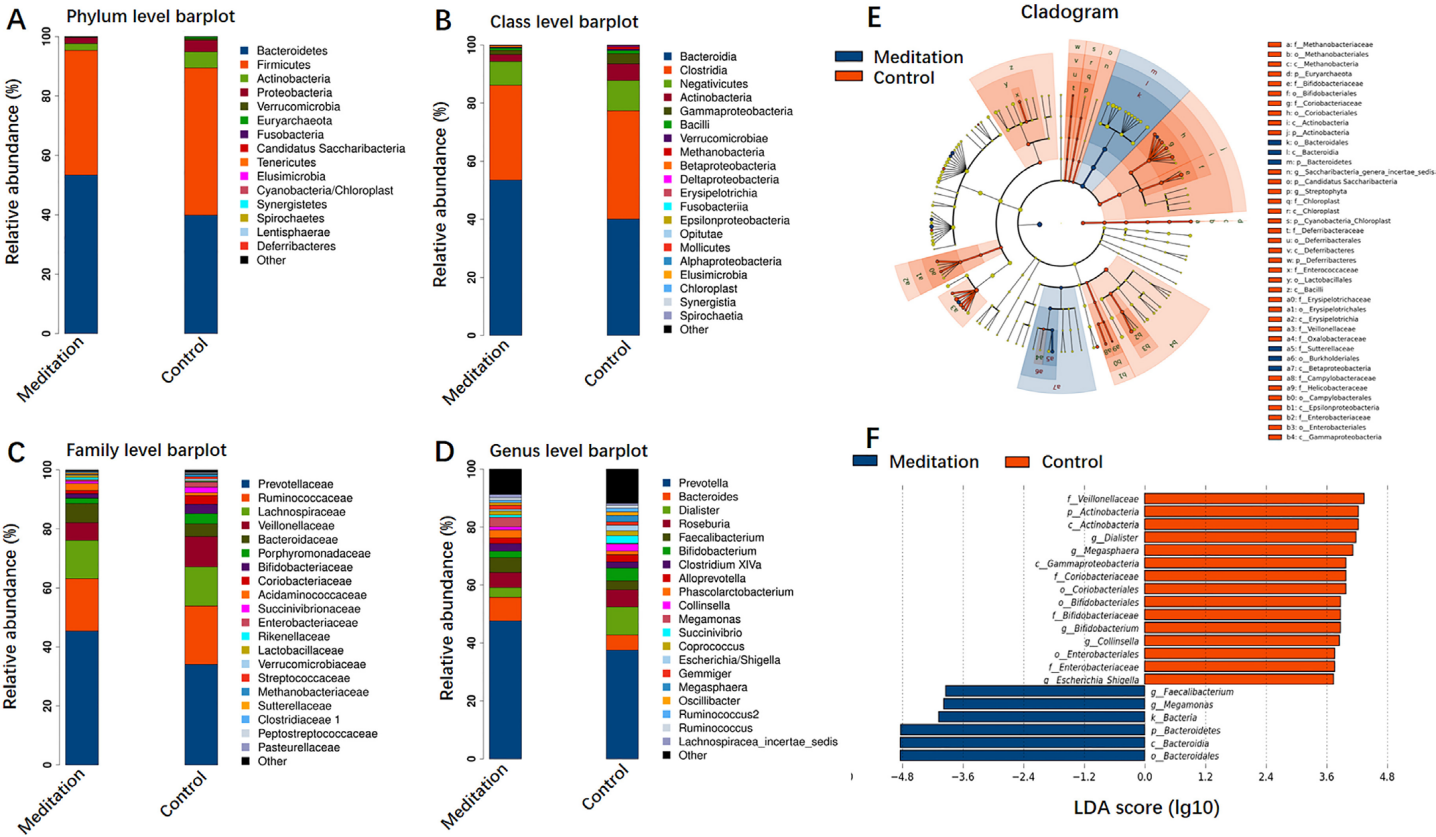
Relative abundances of gut microbiota and linear discriminant analysis (LDA) effect size (LEfSe) in the meditation and control groups. The average composition of bacterial community at the phylum (A), class (B), family (C) and genus (D) levels. (E) Cladogram showing differential bacterial abundance in the meditation (blue) and control (red) groups based on LEfSe analysis. (F) Bar graphs of the LDA score based on LEfSe analysis. LDA score for discriminative features >3.6.

Figure 4A–D shows the composition of bacterial communities at the phylum, class, family and genus levels, respectively. The phyla Bacteroidetes and Firmicutes were dominant in both groups. Bacteroidetes were significantly enriched in the meditation group (figure 4A). At the class level, Bacteroidia, Clostridia and Negativicutes were dominant (figure 4B) and, at the family level, Prevotellaceae, Ruminococcaceae, Lachnospiraceae and Veillonellaceae were significantly enriched in both groups. Prevotellaceae were significantly enriched in the meditation group (figure 4C). At the genus level, *Prevotella*, *Bacteroides*, *Dialister*, *Roseburia* and *Faecalibacterium* were dominant genera in both groups. *Prevotella* and *Bacteroides* were highly enriched in the meditation group with proportions of 42.35% and 6.21% versus 29.15% and 4.07% in the meditation and control groups, respectively (figure 4D).

LEfSe analysis was used to identify the specific bacteria differentially expressed in the meditation and control microbiomes. Compared with the control group, the meditation group showed an increase in the prevalence of Bacteroidales, Sutterellaceae, Burkholderiales and Betaproteobacteria (figure 4E). According to LDA (LDA cut-off score >3.6; p<0.05), two bacterial genera (*Megamonas* and *Faecalibacterium*) were significantly enriched in the meditation group (figure 4F). This exploratory result indicates that meditation could alter the composition of the gut microbiota.

### Functional analysis of gut microbiota and biochemical indices

**Figure 5:**
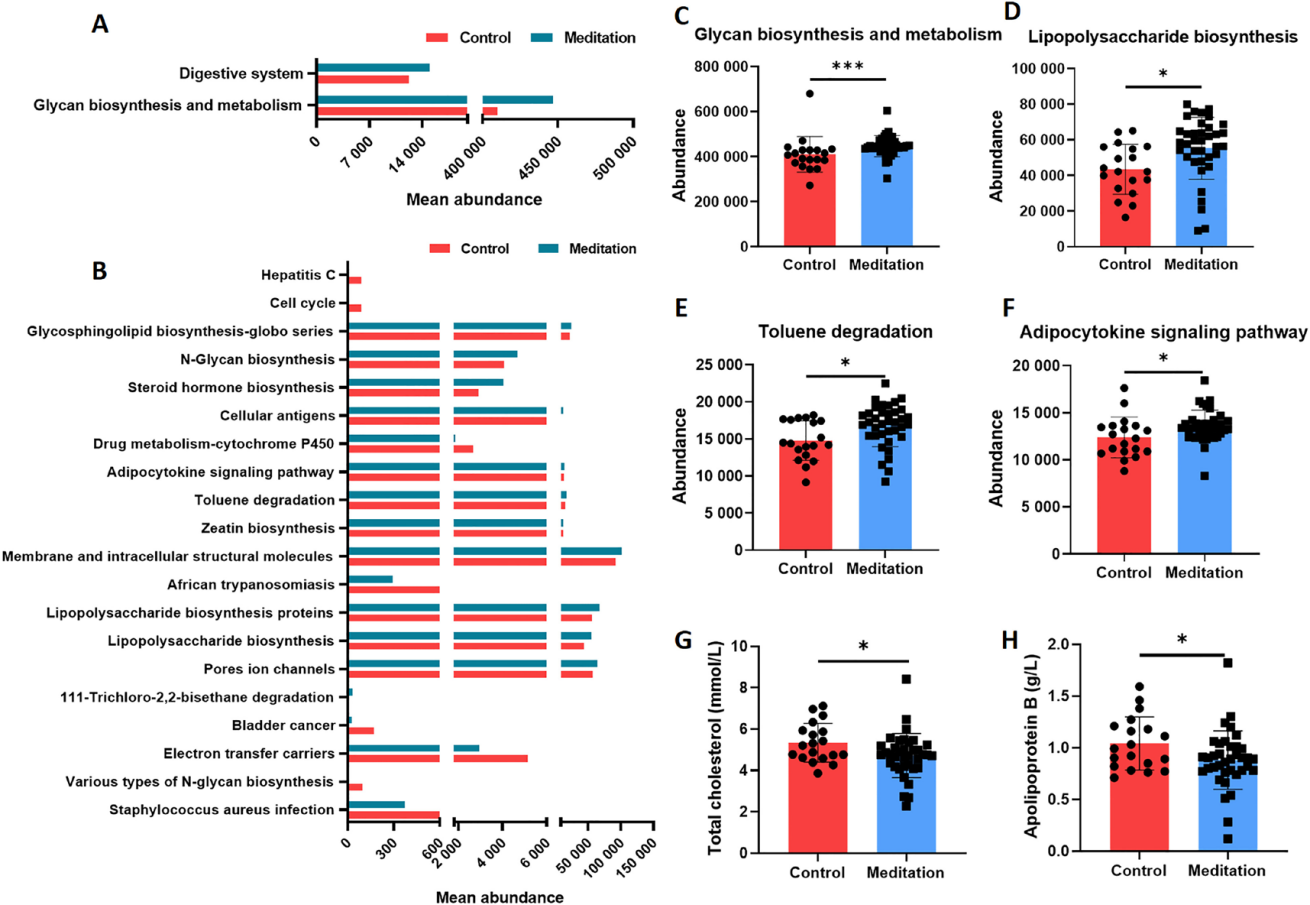
Functional predictions of faecal microbiome and plasma biochemical indices in the meditation and control groups. PICRUSt prediction based on the KEGG annotation at (A) level 2 and (B) level 3 (the top 20 terms were listed and sorted by the p value). (C) Abundance of the glycan biosynthesis and metabolism pathway (Mann–Whitney test, ***p<0.001). (D) Abundance of lipopolysaccharide biosynthesis pathway (unpaired t-test, *p<0.05). (E) Abundance of the toluene degradation pathway (unpaired t-test, *p<0.05). (F) Abundance of the adipocytokine signalling pathway (unpaired t-test, *p<0.05). Plasma biochemical indices including total cholesterol (G) (Mann–Whitney test, *p<0.05) and apolipoprotein B (H) (unpaired t-test, *p<0.05). KEGG, Kyoto Encyclopaedia of Genes and Genomes; PICRUSt, Phylogenetic Investigation of Communities by Reconstruction of Unobserved States.

To explore the altered function of faecal microbiota, we used PICRUSt analysis to infer the abundance of the Kyoto Encyclopaedia of Genes and Genomes (KEGG) pathways at level 2 (figure 5A) and level 3 (figure 5B) using 16S rRNA gene amplicon sequencing. The results revealed significant differences in the abundance of KEGG pathways between the meditation and control groups. Several pathways were significantly enriched in the meditation group, including glycan biosynthesis and metabolism at level 2 and lipopolysaccharide biosynthesis at level 3 (figure 5C–F). Biochemical indices differed significantly between the two groups, including total cholesterol (figure 5G) and apolipoprotein B (figure 5H).

## Discussion

### Main findings

To the best of our knowledge, this is the first study to assess the potential regulation of human gut microbiota by long-term (several years) deep meditation. To minimise the effect of confounding factors, our study strictly matched age, sex, smoking, alcohol consumption and dietary habits between the enrolled monks and control subjects. We observed that the intestinal microbiota composition in the meditation group significantly differed from that of the control group. The control group exhibited higher Chao1, observed-species and Shannon and Simpson indices than the meditation group, indicating the high gut microbial community richness and diversity of the control group. This finding may be related to the sedentary lifestyle of monks (reciting and meditating for long periods each day). ANOSIM and PCoA were used to evaluate the similarity of bacterial communities and to show the significant separation between the meditation and control groups. Altogether, α- and β-diversity indices provided robust evidence that the gut microbiota of the meditation group differed from that of the control subjects.

In this study, we found that several bacterial species differed significantly between the meditation and control groups. At the genus level, *Prevotella*, *Bacteroides*, *Dialister*, *Roseburia* and *Faecalibacterium* were predominant in both groups. *Prevotella* and *Bacteroides* were found to be significantly enriched in the meditation group. *Prevotella* was found to be highly abundant in healthy controls compared with that in patients with major depressive disorder.^16 17^ In addition, the abundance of *Prevotella* was shown to be significantly reduced in samples of autistic children.^18^ In a rat food addiction model, the administration of *Bacteroides uniformis* CECT 7771 was shown to impact the brain reward response, ameliorating binge eating and decreasing anxiety-like behaviour.^19^ Based on the LEfSe analysis, *Megamonas* and *Faecalibacterium* were significantly enriched in the meditation group. *Megamanus* has been associated with all measured psychocognitive traits.^20^ In addition, *Faecalibacterium* was found to be significantly reduced in patients with anxiety disorders compared with healthy controls^21^ and was associated with a higher quality of life,^22^ which is consistent with the findings of our study. Collectively, several bacteria enriched in the meditation group were associated with the alleviation of mental illness, suggesting that meditation can influence certain bacteria that may have a role in mental health.

Glycan biosynthesis, metabolism and lipopolysaccharide biosynthesis pathways were enriched in the meditation group. Glycans can reportedly alleviate intestinal inflammation, improve barrier function and reduce infection-induced colitis.^23^ Studies have shown that the presence of LPS-stimulated Treg cells or exogenous IL-10 significantly promotes IL-10 production by neutrophils and that isoflavone-rich diet anspecifically regulates LPS biosynthesis in the gut microbiota, conferring an anti-inflammatory response and reducing disease severity.^24^ We have previously reported that inflammatory factors are downregulated in monks practising meditation.^6^ Furthermore, the meditation group was significantly enriched in toluene degradation and adipocytokine signalling pathways. These findings show the potential effects of meditation on the nervous system through the intestinal flora. Dysregulation of the adipocytokine signalling pathway has been observed in the depression mouse model. In addition, results from human subjects indicate that anxiety and depression can be correlated with adiponectin levels.^25^ Chronic toluene exposure reportedly increases anxiety in the burying behaviour test of mice, and toluene can reduce neurogenesis and enhance neuronal death.^26^ The results of plasma biochemical indices further illustrate the impact of meditation on human health. We found that total cholesterol and apolipoprotein B levels were higher in the control group, which decreased immune function and increased the risk of cardiovascular disease.^27^ These results suggest that meditation may positively impact psychosomatic conditions.

### Study limitations

The current study has several limitations. Considering the special environment of high-altitude living and hypoxia, the participating subjects may present with diseases which greatly reduces the enrolment ratio of collected samples. The 16S rRNA sequencing method employed in the present study offers no direct data on functionally important changes in the microbiota. In the future, metagenomic sequencing should be undertaken to examine functional changes in the intestinal microbiota.

### Implications

Long-term deep meditation could profoundly impact psychosomatic disorders by altering the structure of the human gut flora. In particular, with the help of a trained therapist, clinicians can provide improved treatment with earlier remission and overall improvements in patients.^28^ Therefore, the effectiveness of meditation in psychosomatic diseases may be a key research avenue in the coming years.

## Conclusion

The intestinal microbiota composition was significantly altered in Buddhist monks practising long-term meditation compared with that in locally recruited control subjects. Bacteria enriched in the meditation group at the genus level had a positive effect on human physical and mental health. This altered intestinal microbiota composition could reduce the risk of anxiety and depression and improve immune function in the body. The biochemical marker profile indicates that meditation may reduce the risk of cardiovascular diseases in psychosomatic medicine. These results suggest that long-term deep meditation may have a beneficial effect on gut microbiota, enabling the body to maintain an optimal state of health. This study provides new clues regarding the role of long-term deep meditation in regulating human intestinal flora, which may play a positive role in psychosomatic conditions and well-being.

## Data Availability

Data are avaliable upon reasonable request.

## References

[1] Ekman P , Davidson RJ , Ricard M , et al . Buddhist and psychological perspectives on emotions and well-being. Curr Dir Psychol Sci 2005;14:59–63. 10.1111/j.0963-7214.2005.00335.x

[2] Ospina MB , Bond K , Karkhaneh M , et al . Meditation practices for health: state of the research. Evid Rep Technol Assess 2007;155:1–263. PMC4780968 17764203

[3] Danhauer SC , Addington EL , Sohl SJ , et al . Review of yoga therapy during cancer treatment. Support Care Cancer 2017;25:1357–72. 10.1007/s00520-016-3556-9 28064385 PMC5777241

[4] Kozasa EH , Tanaka LH , Monson C , et al . The effects of meditation-based interventions on the treatment of fibromyalgia. Curr Pain Headache Rep 2012;16:383–7. 10.1007/s11916-012-0285-8 22717699

[5] Wielgosz J , Goldberg SB , Kral TRA , et al . Mindfulness meditation and psychopathology. Annu Rev Clin Psychol 2019;15:285–316. 10.1146/annurev-clinpsy-021815-093423 30525995 PMC6597263

[6] Xue T , Chiao B , Xu T , et al . The heart-brain axis: a proteomics study of meditation on the cardiovascular system of Tibetan monks. EBioMedicine 2022;80:104026. 10.1016/j.ebiom.2022.104026 35576643 PMC9118669

[7] Yang B , Wei J , Ju P , et al . Effects of regulating intestinal microbiota on anxiety symptoms: a systematic review. Gen Psychiatr 2019;32:e100056. 10.1136/gpsych-2019-100056 31179435 PMC6551444

[8] Cryan JF , Dinan TG . Mind-altering microorganisms: the impact of the gut microbiota on brain and behaviour. Nat Rev Neurosci 2012;13:701–12. 10.1038/nrn3346 22968153

[9] Yano JM , Yu K , Donaldson GP , et al . Indigenous bacteria from the gut microbiota regulate host serotonin biosynthesis. Cell 2015;161:264–76. 10.1016/j.cell.2015.02.047 25860609 PMC4393509

[10] Schirmer M , Smeekens SP , Vlamakis H , et al . Linking the human gut microbiome to inflammatory cytokine production capacity. Cell 2016;167:167. 10.1016/j.cell.2016.11.046 27984736

[11] Kennedy PJ , Cryan JF , Dinan TG , et al . Kynurenine pathway metabolism and the microbiota-gut-brain axis. Neuropharmacology 2017;112:399–412. 10.1016/j.neuropharm.2016.07.002 27392632

[12] Grochowska M , Wojnar M , Radkowski M . The gut microbiota in neuropsychiatric disorders. Acta Neurobiol Exp 2018;78:69–81. 10.21307/ane-2018-008 30019700

[13] Jia W , Zhen J , Liu A , et al . Long-term vegan meditation improved human gut microbiota. Evid Based Complement Alternat Med 2020;2020:9517897. 10.1155/2020/9517897 32714427 PMC7358775

[14] Segata N , Izard J , Waldron L , et al . Metagenomic biomarker discovery and explanation. Genome Biol 2011;12:R60. 10.1186/gb-2011-12-6-r60 21702898 PMC3218848

[15] Langille MGI , Zaneveld J , Caporaso JG , et al . Predictive functional profiling of microbial communities using 16S rRNA marker gene sequences. Nat Biotechnol 2013;31:814–21. 10.1038/nbt.2676 23975157 PMC3819121

[16] Chung Y-CE , Chen H-C , Chou H-CL , et al . Exploration of microbiota targets for major depressive disorder and mood related traits. J Psychiatr Res 2019;111:74–82. 10.1016/j.jpsychires.2019.01.016 30685565

[17] Wu J , Li J , Gaurav C , et al . CUMS and dexamethasone induce depression-like phenotypes in mice by differentially altering gut microbiota and triggering macroglia activation. Gen Psychiatr 2021;34:e100529. 10.1136/gpsych-2021-100529 34970638 PMC8671983

[18] Kang D-W , Park JG , Ilhan ZE , et al . Reduced incidence of Prevotella and other fermenters in intestinal microflora of autistic children. PLoS One 2013;8:e68322. 10.1371/journal.pone.0068322 23844187 PMC3700858

[19] Agustí A , Campillo I , Balzano T , et al . Bacteroides uniformis CECT 7771 modulates the brain reward response to reduce binge eating and anxiety-like behavior in rat. Mol Neurobiol 2021;58:4959–79. 10.1007/s12035-021-02462-2 34228269 PMC8497301

[20] Renson A , Kasselman LJ , Dowd JB , et al . Gut bacterial taxonomic abundances vary with cognition, personality, and mood in the Wisconsin Longitudinal Study. Brain Behav Immun Health 2020;9:100155. 10.1016/j.bbih.2020.100155 34589897 PMC8474555

[21] Wang Z , Liu S , Xu X , et al . Gut microbiota associated with effectiveness and responsiveness to mindfulness-based cognitive therapy in improving trait anxiety. Front Cell Infect Microbiol 2022;12:719829. 10.3389/fcimb.2022.719829 35281444 PMC8908961

[22] Valles-Colomer M , Falony G , Darzi Y , et al . The neuroactive potential of the human gut microbiota in quality of life and depression. Nat Microbiol 2019;4:623–32. 10.1038/s41564-018-0337-x 30718848

[23] Kelm M , Quiros M , Azcutia V , et al . Targeting epithelium-expressed sialyl Lewis glycans improves colonic mucosal wound healing and protects against colitis. JCI Insight 2020;5:e135843. 10.1172/jci.insight.135843 32427587 PMC7406298

[24] Ghimire S , Cady NM , Lehman P , et al . Dietary isoflavones alter gut microbiota and lipopolysaccharide biosynthesis to reduce inflammation. Gut Microbes 2022;14:e2127446. 10.1080/19490976.2022.2127446 PMC9542810 36179318

[25] Wilhelm CJ , Choi D , Huckans M , et al . Adipocytokine signaling is altered in Flinders sensitive line rats, and adiponectin correlates in humans with some symptoms of depression. Pharmacol Biochem Behav 2013;103:643–51. 10.1016/j.pbb.2012.11.001 23153628 PMC4408933

[26] Paez-Martinez N , Flores-Serrano Z , Ortiz-Lopez L , et al . Environmental enrichment increases doublecortin-associated new neurons and decreases neuronal death without modifying anxiety-like behavior in mice chronically exposed to toluene. Behav Brain Res 2013;256:432–40. 10.1016/j.bbr.2013.09.007 24012598

[27] Shen H , Chen M , Cui D . Biological mechanism study of meditation and its application in mental disorders. Gen Psychiatr 2020;33:e100214. 10.1136/gpsych-2020-100214 32695961 PMC7359050

[28] Sun L , Xu M , Shi Y , et al . Decoding psychosis: from national genome project to national brain project. Gen Psychiatr 2022;35:e100889. 10.1136/gpsych-2022-100889 36248024 PMC9511649

